# Observations on the Climate of New Zealand

**Published:** 1840-04-01

**Authors:** 


### Observations on the Climate of New Zealand. By *W. Swainson*, Esq. Octavo, pp. 80. Smith and Elder. 1840.

The tide of emigration, from the mother country to various remote regions, now runs so strong, that medical men are daily called upon to give information as to the climates of those countries to which so many of our countrymen and women are forced to expatriate themselves, from the difficulty they experience in retaining *caste* in the social scale. This difficulty is well known to depend on exuberance of population, and its inevitable consequence, paucity of eligible occupation. The current of colonization has recently set strong towards New Zealand—a country which, from its insular character, climate, and fertility, is calculated to become a valuable addition to British dependencies.

The improvement of human life, as to duration, has added to the difficulties of providing for our offspring! One hundred years ago, the deaths were about four per cent. of the population. They are now about two per cent. Or, in other words, the probability of life has doubled in a century. This amelioration can have had but little connexion with climate—for in this respect there could not have been any material change in England during the last century. But still climate must have considerable influence on man, especially as to the production of certain diseases, as phthisis for instance. The mortality resulting from this disease is computed to be about 25 per cent. in England, Austria, Prussia, Switzerland, and Belgium—23 in France—17 in St. Petersburg and New York—while in some parts of Africa, the disease is almost unknown. There can be little doubt that *early* removal to a climate free from great and sudden vicissitudes of temperature, has, in many instances, arrested or retarded the progress of tubercles. In most of the countries, however, to which our phthisical invalids are sent, the disease is as frequent as in England.

"Assuming the accuracy of our information, and comparing their several mean temperatures, the climate of the southern coast of England, in the most essential qualities, is not inferior to that of any of the most celebrated places of resort in Europe. The mean temperature for the whole year is certainly somewhat lower, and its vicissitudes may be more frequent in England; but what is, perhaps, more important, these vicissitudes are more limited in their range—

the general temperature is more *equable*. The temperature is highest in Devonshire and Cornwall, the annual mean being 52° 50′; in the Winter, 44° 50′; in the Spring, 49° 60′; in Summer, 60° 50′; and in the Autumn, 63° 50′. While in the northern counties the mean for the year is only 47°; Winter, 37° 50′; Spring, 44° 50′; Summer, 59° 50′; Autumn, 48° 65′.

In variability and uncertainty, so far at least as is indicated by thermometrical observations, the advantage is decidedly in favour of the climate of the more favoured parts of England. At Penzance, and along the coasts of Cornwall and Devonshire, the mean *range* during the twenty-four hours is only 8°; while at Naples, the daily range is 13° 5′; at Rome, 11°; at Montpelier, 12°. It varies also considerably with the season of the year; being much greater in England in June than in January.

The annual *range* of the thermometer in the south-eastern division of the kingdom is about 67°, and still more limited in the south-west coast, being little more than 50°; but even in the former, considerably less than in Nice, Naples, and the most reputed continental places of resort." 19.

"But the difference in favour of England appears most striking, when we compare the mean temperature of their coldest and hottest months, which in Cornwall is only about 18°, almost double in the South-east of France; 31° in the South-west of France; and 32° in Italy. We cannot be surprised, therefore, at the condemnation of the South of France and the North of Italy, by Crichton, Portal, Morgan, Hawkins, and Johnson. In point of general salubrity, there can be no comparison between those parts of the Continent usually resorted to, and our own country. The duration of life in the least healthy districts of England exceeds considerably that of the most favoured parts of the Continent, and that very disease, once considered almost peculiar to this country, being in fact no less prevalent in the latter than the former. In the time of Celsus even, we find a removal *from* Italy recommended. 'Quod si mali plus est, et vera phthisis, inter initia protinus occurrere necessarium est; neque enim facile is morbus cum inveteraverit, evincitur. Opus est si vires patiuntur, longa navigatione, cœli mutatione, sic ut densius quam id est ex quo discedit æger, petatur: idioque aptissime Alexandriam ex Italia itur.'" 20.

As tubercular disease is nearly unknown in Egypt, the general character of the climate becomes a matter of interest. It has been described by competent authority, to be at all times extremely damp (at least about Alexandria)—the atmosphere being saturated with saline vapour, which condenses on the walls of the houses in small crystals of nitre, common salt, and muriate of ammonia. The soil is everywhere coated with these saline particles, yet the constant breathing of such an atmosphere does not appear to injure the constitution. It is asserted that no instance of consumption has been seen among the Arabs.

In the Channel Islands phthisis is believed to be much less common than in England—and the peculiar dampness of these islands is very similar to that of Alexandria.

"Besides its insular position (says Dr. Scholefield) the island of Jersey is everywhere thick-set with wood, an imperfect system of drainage or no drainage at all, the prevalence of winds from the south-west blowing directly up the channel, from across the Atlantic Ocean, impregnated with moisture and saline particles that cause the moisture to be more slowly dissipated into vapour, the great secession of the tide at low water, leaving all around the island a vast extent of bare rocks and sands, from which saline vapours largely arise, in consequence of the comparative warmth of the temperature even during the Winter months, while these vapours are scattered in all directions by currents of air arising from the great diversity of the island in hill and dale." 21.

With reference then to the influence of climate, in the temperate zone at least, it would seem that the circumstances which characterize their anti-phthisical qualities are a mild equable atmosphere—or limited in its range—holding in suspension a considerable quantity of saline moisture.

But to return to the immediate subject before us. Looking at the geographical situation of New Zealand, and in the absence of all practical and direct information—

"If we were told that it consists of a group of islands in the South Pacific Ocean extending from the 34th to the 48th degree of South latitude, and from the 166th to the 179th degree of East longitude, at a distance of twelve hundred miles from the nearest main land, the Continent of New Holland, and having an undulating, and in many places a mountainous surface, we should be led to expect a climate 8° or 10° cooler than that of the South of Portugal in the corresponding latitude of the opposite hemisphere—moderate in its extremes of temperature—of a humid atmosphere—with frequent changes of weather, and in general mild, equable, and showery." 28.

By almost all writers the inhabitants of these islands are described as tall, active, well made, and intelligent. Vegetation is described as luxuriant. The hills are one continued forest of lofty trees, flourishing with superior vigour, and affording most picturesque scenery. The fruits and vegetables that can only be cultivated with success, in equable and temperate weather in Europe, thrive remarkably in New Zealand.

Of the various accounts given by voyagers, those of Captain Cook, and, 70 years afterwards, of Mr. Yate, a missionary, are the most correct, as regards *climate*, because these two had seen the whole course of the seasons throughout the year. We shall take that of Mr. Yate.

"North of the Thames," he observes, "snows are unknown, and frosts are off the ground by nine o'clock in the morning. The country during six months in the year, is subject to heavy gales from the East and North-east, which generally last for three days, and are accompanied with tremendous falls of rain. These gales generally commence in the East, and gradually haul round to the North-west, where they terminate in a violent gust, almost approaching to a hurricane; the clouds then pass away, and the westerly wind blows again with some violence. In the Winter season, the moon rarely either changes or wanes without raising one of these tempestuous gales; and during the whole year, the wind is sure to blow, though it may be only for a few hours, every fall and change of the moon."

"The Spring and Autumn," he continues, "are delightfully temperate, but subject to showers from the West-south-west. Indeed, however fine the Summer may be, we are frequently visited by refreshing rains, which give a peculiar richness to the vegetation, and fertility to the land. The prevailing winds are from South-west to North-west, which within this range, blow upwards of nine months in the year; more frequently the wind is due west. During five months, sea breezes set in from either coast, and meet each other half way across the island." 37.

Compared with the climate of New South Wales, Mr. Earle (1827) remarks that, "though situated in the same latitude as Sydney, we found the climate of New Zealand infinitely superior. Moderate heats and beautiful clear skies succeeded each other every day—free from those oppressive feverish heats which invariably prevail in the middle of the day at Sydney, and from those hot pestilential winds which are the terror of the inhabitants of New South Wales."

"Of the summer temperature it will be observed we have only general statements, but it appears that in the Northern division of the country (that nearest the equator) where these observations were taken, the summer, or rather the three warmest months, (for the summer comprises at least three-fourths of the year), are somewhat hotter than in England, and that the spring and autumn have the temperature, and settled weather of a fine English summer, and that the three coldest months have the wet, broken, tempestuous weather of an English March, but with the temperature of our October." 54.

In comparison with Madeira, the mean temperature is rather higher there than in New Zealand, but the range not more limited. With respect to the influence of the New Zealand climate on European Constitutions our information is not complete. Capt. Cook remarks:—

"Our people," he says, "who were daily exposed to the rain, felt no ill effects from it; on the contrary, such as were sick and ailing when we came in, recovered daily, and the whole crew soon became strong and vigorous, which can only be attributed to the healthiness of the place and the fresh provisions it afforded." An account confirmed by Mr. Yate, who observes that, "those who come here sickly are soon restored to health, the healthy become robust, and the robust fat."

Mr. Earle appears to have noticed the healthy appearance of the missionaries and their families. Giving an account of a visit to one of their stations, he remarks, "the chubby children who peeped at us from all corners, and the very hearty appearance of their parents, plainly evidenced that theirs was an excellent and thriving trade." 56.

Dr. Lang, Captain Fitzroy, Mr. Watkins, and others, give similar evidence. Nevertheless it is proved by evidence that scrofula and consumption are prevalent among the natives of those islands.

"'Consumption, violent rheumatism, and sore eyes,' says Major Cruise, 'seem to be the prevailing diseases of the New Zealanders.' Mr. Earle remarks that, considering the nature of the climate, 'it seems unaccountable that the New Zealanders should be subject to so fatal a disease as galloping consumption.' In his examination before the committee, Mr. Watkins speaks of scrofula as one of the prevalent diseases. 'Their diseases are something like our diseases, with the exception of scrofula being, perhaps, more abundant there.' Mr. Tawell, too, in his evidence, states, 'that scrofula is the prevalent disease among the natives.'" 59.

But it does not follow that a climate should be unhealthy because scrofula is prevalent. It has been seen that England and several other climates, widely different in their nature, are nearly on a par in this respect. Climate, in fact, is only one of the many causes of scrofula and consumption. All uncivilized nations suffer from first intercourse with civilized people. Captain Cook tells us that, when he first visited New Zealand, there was no such thing as disease there. The introduction of Syphilis soon changed the scene! Mr. Watkins stated to the Committee in Parliament, that not one in fifty women were free from the disease in New Zealand! The change of their occupations, too, from rearing pigs and poultry, to felling timber in the woods, has had a very deleterious effect on their health. Rum and tobacco have aided syphilis, and the habit of placing the sick in the open air, exposed to winds and rains, has swelled the ratio of mortality.

The following extract will shew what extreme vicissitudes of temperature occur in New Holland, whose climate, generally, is very salubrious.

"Dr. Lang, in his work on New South Wales, after describing the beauty of the climate for at least eight months in the year, alludes to the occurrence of sudden variations in the temperature, 'The most singular phenomena,' says he, 'in the meteorology of New South Wales, is the occasional prevalence of hot winds from the North-westward. These winds occur on an average four times every Summer, and blow from twenty-four to thirty-six hours each time, the atmosphere all the while feeling like a current of heated air from a furnace, and the thermometer generally standing at from 90° to 100° of Fahrenheit. It has even stood as high on one occasion within my own experience as 112½°.' * * * 'When the hot wind has spent its strength it is usually succeeded instantaneously by a violent gust from the southward, which immediately envelopes the town of Sydney in a whirlwind of dust. I have observed the hot wind terminate instantaneously in a hail-storm of a few minutes' duration from the South-westward, which of course caused the mercury in the thermometer to descend with surprising velocity, the difference of elevation, after a short interval, being on one occasion, when the wind had been unusually hot, not less than 40°.'" 70.

Ophthalmia, dysentery, and influenza are the three most prevalent complaints in New South Wales. The monthly mean temperature of the last-mentioned place and New Zealand are very nearly the same.

The following extract from the Journal of an Invalid, bears on the climate of Van Diemen's Land, which differs considerably from that of Australia.

"'Our Summer days are most uncertain; on Tuesday the thermometer was 92°, with a real hot wind; on Friday it was 52°, with rain, and we were glad to have a roasting fire. From season to season, the changes are less extreme. There is little or no snow in Winter; the climate is dry. But let those withered Indian frames, which a whistle of the wind almost blows to atoms, beware of Van Diemen's Land. The breezes here are generally gales; and when they come from the South, which is almost daily, they are more cutting and cold than the easterly puffs we used to dread across Brunswick Square, London. * * * 'During one hundred and thirty days that I kept a meteorological journal, from October 16th to February 23rd (the Summer in Van Diemen's Land) there were—

| | |
|---|---|
| Rainy days .. .. .. .. | 42 |
| Strong winds .. .. .. .. | 24 |
| Fine and pleasant .. .. .. | 28 |
| Very fine .. .. .. .. | 29 |
| Wind too hot .. .. .. | 7 |

Hottest day, 25th of January, thermometer 99½° in the shade; coldest day, 16th October, thermometer 56°.' It is obvious then, that in point of *equability*, at least, the climate of New Zealand has a decided advantage over that of the neighbouring Australian Colonies." 74.

From the extracts which we have made from this pamphlet, and what we have ourselves learnt from some of our medical brethren who have visited New Holland and New Zealand, we are inclined to think that the climate of the *latter* is little *inferior* to some of the boasted resorts of invalids in Europe. The great advantage of New Zealand over a residence in Italy, Madeira, or other foreign country, will be obvious from the following sensible reasons adduced by Mr. Swainson.

"A mere voyage to Madeira, and a temporary sojourn there is within the means of few; a permanent residence, almost entirely out of the question. Admitting it, therefore, to possess all the advantages claimed for it by its warmest advocates, it is practically valueless to the British public.

Not so, however, New Zealand—an English Colony, peopled by our own countrymen, the greater number from the most respectable class of society, and carrying with them English habits, customs, comforts, and conveniencies; from its insularity, position, and natural productions, calculated to afford a wide and profitable field for every branch of commercial enterprize, it appears to present to the people of this country a *sanative station* accessible to all classes of the community." 77.

The author of this pamphlet is not a medical man: but he is evidently imbued with a philosophic spirit, accustomed to weigh evidence with accuracy, and draw inferences with great caution. We return him many thanks for the pleasure and instruction we have derived from a perusal of his Essay.

